# Disrupted topological organization of functional brain networks in Alzheimer’s disease patients with depressive symptoms

**DOI:** 10.1186/s12888-022-04450-9

**Published:** 2022-12-20

**Authors:** Zhongwei Guo, Kun Liu, Jiapeng Li, Haokai Zhu, Bo Chen, Xiaozheng Liu

**Affiliations:** 1 Tongde Hospital of Zhejiang Province, Zhejiang Provincial Health Commission, Hangzhou, 310012 China; 2 The Second Affiliated Hospital and Yuying Children’s Hospital, Wenzhou Medical University, Wenzhou, Zhejiang 325027 China; 3 The Second Clinical Medical College, Zhejiang Chinese Medicine University, Hangzhou, 310000 China

**Keywords:** Alzheimer’s disease, Depression, Functional brain network, Graph theory analysis

## Abstract

**Background:**

Depression is a common symptom of Alzheimer’s disease (AD), but the underlying neural mechanism is unknown. The aim of this study was to explore the topological properties of AD patients with depressive symptoms (D-AD) using graph theoretical analysis.

**Methods:**

We obtained 3-Tesla rsfMRI data from 24 D-AD patients, 20 non-depressed AD patients (nD-AD), and 20 normal controls (NC). Resting state networks were identified using graph theory analysis. ANOVA with a two-sample *t*-test post hoc analysis in GRETNA was used to assess the topological measurements.

**Results:**

Our results demonstrate that the three groups show characteristic properties of a small-world network. NCs showed significantly larger global and local efficiency than D-AD and nD-AD patients. Compared with nD-AD patients, D-AD patients showed decreased nodal centrality in the pallidum, putamen, and right superior temporal gyrus. They also showed increased nodal centrality in the right superior parietal gyrus, the medial orbital portion of the right superior frontal gyrus, and the orbital portion of the right superior frontal gyrus. Compared with nD-AD patients, NC showed decreased nodal betweenness in the right superior temporal gyrus, and increased nodal betweenness in medial orbital part of the right superior frontal gyrus.

**Conclusions:**

These results indicate that D-AD is associated with alterations of topological structure. Our study provides new insights into the brain mechanisms underlying D-AD.

**Supplementary Information:**

The online version contains supplementary material available at 10.1186/s12888-022-04450-9.

## Introduction

Depression is one of the major psychobehavioral symptoms in Alzheimer’s disease (AD). It increases the difficulty of interventions and may lead to death [1]. Understanding the pathogenesis of depression associated with AD will be helpful in discovering effective therapies and early interventions.

A few studies of functional magnetic resonance imaging (fMRI) have shown that changes of brain function in multiple brain regions are involved in the pathogenesis of depression in AD. These studies adopted several analysis methods, including amplitude of low frequency fluctuations (ALFF) [2, 3], functional connectivity (FC) [4, 5], and degree centrality (DC) [6]. Mu et al. [2] reported lower ALFF in the bilateral superior frontal gyrus, left middle frontal gyrus, and the left inferior frontal gyrus, in depressed AD patients (D-AD) compared with non-depressed AD patients (nD-AD). Our previous studies also showed that D-AD patients had increased FC between amygdala and orbitofrontal cortex, and decreased FC among amygdala, medial prefrontal cortex, and inferior frontal gyrus [4]. Furthermore, we reported lower DC in the right middle frontal, precentral, and postcentral gyrus [6]. The above studies show that depression in AD is associated with dysfunctional neural activity in multiple brain regions. Several studies have also shown that neuronal connections undergo functional changes in D-AD patients. Using diffusion tensor imaging, Yatawara et al. [7] reported reduced tract integrity of right hemisphere subcortical and the corpus callosum geniculate in depressed patients with mild AD. When compared with nD-AD patients, D-AD patients showed significantly increased mean diffusivity and radial diffusivity in the bilateral cingulum bundle (CB) and right uncinate fasciculus (UF). These results suggest that myelin injury in the bilateral CB and right UF might contribute to the pathophysiology of depressive symptoms in AD [8]. The aforementioned studies strongly suggest that the regulation of depression in AD patients involves several brain circuits, including the emotional circuit [9], the default mode network [10], and the sensorimotor network [11]. However, the methodological approaches adopted by previous studies did not assess the complexity of regional interactions at the level of the entire brain network. To overcome this limitation and gain a more comprehensive understanding of the neural mechanisms associated with depression in AD, we explore the topological organization of intrinsic brain networks on a large scale that encompasses the entire structure.

Graph theory has become popular for describing the characteristics of brain neural networks. In this approach, networks are represented graphically via global network parameters and regional nodal parameters [12]. Using specific graph measures, it is possible to characterize functional specialization and integration of the brain as a network. Small-worldness is a metric that reflects the optimal balance between network separation and consolidation. Global efficiency is a scalar measure of information flow, defined as the inverse of all shortest path lengths in a given network. Local efficiency and global efficiency are calculated similarly, but the former is computed at the level of individual nodes rather than the entire network. For a given node, nodal degree is the number of neighbors connected to it, which reflects the importance of the node within the network. Betweenness centrality indicates the ability to connect between different nodes connected to a given node [12]. Using graph theory, some studies have concluded that human brain networks are organized according to special principles [13, 14]. Furthermore, disruption of network organization is associated with several neuropsychiatric disorders, such as AD and major depressive disorder (MDD) [15–19]. Graph theory has been used to expose disruption of large-scale brain network integrity in AD [15–17]. He et al. [15] measured reduced overlap between network nodes in AD patients, which led to alterations of the small-world property. Sanz-Arigita et al. [16] also found slower information transmission in brain networks of AD patients, with values approaching those of theoretical random networks. With regard to nodal properties, AD patients had reduced overlap within the bilateral hippocampus compared with a healthy control population [17]. Previous studies have shown that patients with MDD exhibit a small-world architecture like healthy individuals, which suggests the presence of a functional balance between segregation and integration of brain networks. Compared with normal controls (NC), MDD subjects present significant differences in local network measures for many brain regions, mainly located in the cognitive control network (CCN) and default mode network (DMN) [18, 19]. Furthermore, altered nodal centrality in basal ganglia correlated with both disease duration and severity of MDD [19]. Recently, Serra et al. [20] investigated the alterations of brain network topology in AD patients with neuropsychiatric symptoms (NPS). They found that, compared with AD patients that had no NPS, those with NPS showed disconnection within a subnetwork involving mainly temporal and cerebellar nodes. However, their patient group included patients with amnestic cognitive impairment in addition to AD patients, and they did not separately analyze brain network topology in AD patients with depression symptoms.

In this study we utilize resting-state functional magnetic resonance imaging (rsfMRI), and we apply graph theory to investigate alterations in the topological structure of brain networks. We directly compare D-AD patients with nD-AD patients and with normal controls (NC). We used the automated anatomical labeling (AAL) template to divide the brain into 90 regions of interest, across which we explored the altered topological organization of intrinsic functional brain networks in D-AD patients [16–20].

## Materials and methods

### Patients

Our study involved 20 nD-AD patients, 24 D-AD patients, and 20 NC. Inclusion and exclusion criteria for probable AD were similar to those of previous studies [3, 5].

The diagnosis of depression followed the fourth edition of the Diagnostic and Statistical Manual of Mental Disorders [21]. The severity of depression was rated using Hamilton Depression Rating Scale (HAMD) [22] and Neuropsychiatric Inventory (NPI) [23]. We defined inclusion criteria as a HAMD score between 7 and 17 and a D-NPI score ≥ 4, indicating clinically significant symptoms [24].

### MRI scan

All subjects underwent a functional fMRI scan on a 3-T Siemens scanner (Siemens Magnetom Verio; Siemens Medical Systems, Erlangen, Germany). Scanning parameters were similar to those adopted in our previous studies [3, 5]. T2^*^-weighted echo planar images were acquired with the following parameters: 33 axial slices, thickness/gap = 4.8/0 mm, matrix size = 64 × 64, TR/TE = 2000/30 ms, flip angle = 90°, and field of view (FOV) = 200 mm × 200 mm. Each functional imaging run consisted of 200 volumes. For registration purposes, a high-resolution T1-weighted structural image was acquired from each participant using the following parameters: inversion time/repetition time/echo time (TI/TR/TE) = 900/1900/2.48 ms, flip angle = 9°, 128 slices, FOV = 256 mm × 256 mm, resolution = 1 mm × 1 mm × 1 mm.

### Data processing

Data preprocessing was performed using SPM8 (http://www.fil.ion.ucl.ac.uk/spm) and Data Processing Assistant for Resting State fMRI v2.3 (http://www.restfmri.net). After removal of the first 10 volumes to allow for scanner equilibration effects, we carried out the following preprocessing steps: correction for slice-timing and head motion, spatial normalization to Montreal Neurological Institute (MNI) space with a resampled resolution of 3 mm × 3 mm × 3 mm using T1 Unified Segmentation, spatial smoothing with a 6-mm Gaussian kernel along all three directions, linear trend removal, and temporal bandpass filtering (0.01–0.08 Hz). Finally, we used T1 images from each subject to define cerebrospinal fluid and white matter in native space, in order to calculate interference regressors for white matter and cerebrospinal fluid as well as head motion parameters. Nuisance signals were further removed from the resulting images by regressing out head motion parameters of Friston 24, cerebral spinal fluid signal, and white-matter signal. With regard to motion correction, participants never exceeded a 2.5-mm displacement along any axis, and an angular motion of 2.5 for the entire duration of the rsfMRI scan [3, 5].

### Head motion analysis

Head movement during scanning may impact the results. To take this factor into account, we calculated the mean relative root mean square and mean framewise displacement power (FD) of head movement [25] and performed ANOVA on the head movement parameters of the three groups to compare differences across groups.

### Brain network construction

We divided the whole brain into 90 regions of interest according to the AAL atlas, with each ROI representing a node. We utilized the residual images after data preprocessing to calculate the cross-correlations between all possible pairs of 90 nodes, forming a 90 × 90 matrix of correlation coefficients for each subject [26].

We constructed functional brain networks over the whole range of costs (0.05–0.40) at an interval of 0.01 using a weighted matrix. We calculated graph measures across this threshold range using functions implemented in the GRETNA software package [26]. We then measured the resulting regional nodal characteristics (nodal centrality) of the network, including nodal degree, nodal efficiency, and nodal betweenness centrality. We used small-worldness and network efficiency parameters (local efficiency and global efficiency) as global network measures [26].

### Statistical analysis

We initially performed Jarque-Bera tests on demographic and clinical characteristics of the participants to assess normality of the underlying data distribution. We then assessed demographic and clinical characteristics of three groups using ANOVA and χ^2^ tests.

For all network measures (including small-worldness, local efficiency, global efficiency, nodal degree, nodal efficiency, and nodal betweenness centrality), we computed separately the area under the curve (AUC) across the full range of sparsity thresholds. We then compared the AUC values across NC, nD-AD, and D-AD groups. First, we used ANCOVA to identify potential differences in topological measurements across the three groups. We then performed two-by-two post hoc analyses using two-sample *t*-tests in GRETNA [26]. We used age, sex, and mean relative displacement of head motion as covariates in the statistical analysis, to reduce their possible effects on the data. We adopted a significance level of *P* < 0.05 for all tests and applied Bonferroni correction for multiple comparisons. We assessed the relationship between topological measurements and MMSE, NPI, and HAMD scores in both nD-AD and D-AD patients using Pearson’s correlation.

## Results

### General clinical data

The three groups did not differ significantly with regard to age (*F* = 1.720, *P* = 0.188), sex distribution (χ^2^ = 6.000, *P* = 0.199), or years of education (*t* = 1.070, *P* = 0.348). D-AD and nD-AD groups did not differ with regard to duration of disease (*t* = 0.164, *P* = 0.9699). However, we found significant differences across groups with regard to MMSE, HAMD, and NPI scores (*F* = 101.8, *P* < 0.001; *F* = 190.0, *P* < 0.001; *F* = 238.0, *P* < 0.001, respectively). D-AD and nD-AD groups did not differ with regard to MMSE (*t* = 0.774, *P* = 0.446), however they showed significant differences in HAMD and NPI scores (*t* = − 13.044, *P* < 0.001; *t* = − 17.701, *P* < 0.001, respectively). D-AD and NC groups did not differ with regard to HAMD and NPI (*t* = 0.200, *P* = 0.295; *t* = 0.222, *P* = 0.189). ANOVA analysis did not return any significant difference in mean motion among the three groups. The *F* and *p* values for the relative root mean square were 0.300 and 0.745, respectively. For the mean FD, values were *t* = 0.77 and *p* = 0.467. Table 1 lists relevant details.

**Table 1:** Demographics and neuropsychological data

	D-AD	nD-AD	NC	F/χ^2^	*p*
Gender, n (M/F)	24(17/7)	20(10/10)	20(11/9)	6.000	0.199
Age, years	71.2 ± 5.3	74.2 ± 5.5	70.8 ± 3.3	1.720	0.188
Duration (m)	15.7 ± 10.2	14.8 ± 7.4	–	121.0	0.000
Education, years	9.7 ± 2.5	9.0 ± 2.2	8.7 ± 2.1	1.070	0.348
MMSE	20.5 ± 2.9	20.0 ± 2.5	29.1 ± 0.8	101.8	0.000
HAMD	12.7 ± 2.5	3.35 ± 1.9	1.30 ± 0.9	190.0	0.000
D-NPI	5.82 ± 1.5	1.21 ± 0.51	0.60 ± 0.6	238.0	0.000
RMS of head motion	0.08 ± 0.06	0.08 ± 0.08	0.06 ± 0.03	0.30	0.745
FD of head motion	0.29 ± 0.18	0.28 ± 0.15	0.24 ± 0.12	0.77	0.467

Data represent mean ± SD. Data were analyzed using independent-samples *t*-tests. AD = Alzheimer’s disease; D-AD = AD with depression; nD-AD = non-depressed AD patients. M = Male; F = Female; MMSE = Mini-Mental State Examination; D-NPI = depression domain on Neuropsychiatric Inventory; HAMD = Hamilton Depression Rating Scale; RMS, root mean square.

### Global properties

**Fig. 1:**
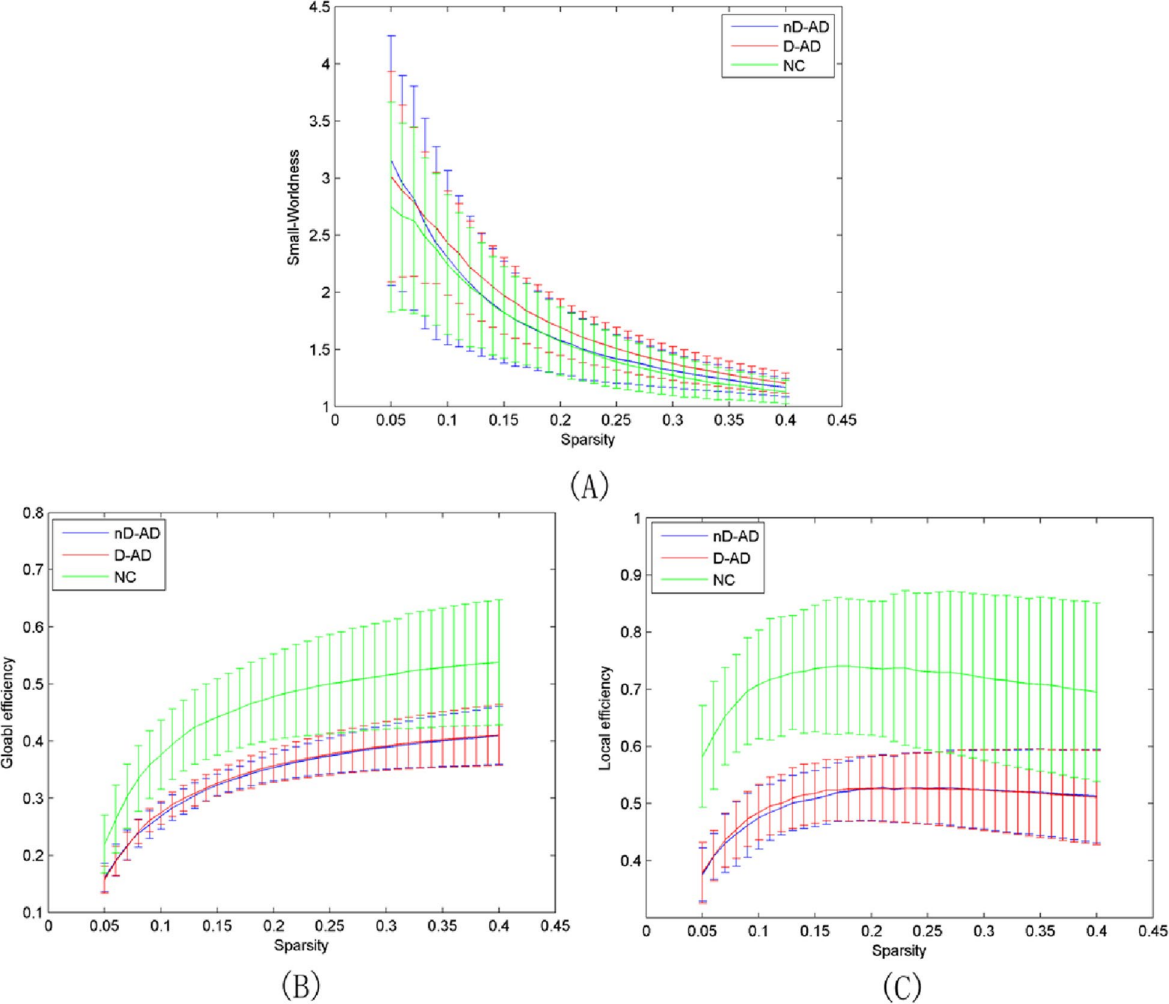
Global network properties of D-AD and nD-AD patients for different sparsity thresholds. (A) Small-worldness; (B) network global efficiency; (C) network local efficiency. The three groups (D-AD, nD-AD, and NC) show properties that are typical of small-world networks. Global network properties are similar between AD patients with depression and those without depression. The NC groups presents significantly larger global and local efficiency than D-AD and nD-AD groups. nD-AD, non-depressed AD patients; D-AD, depressed AD patients; NC, normal controls

D-AD and nD-AD groups show significantly lower global and local efficiency than the NC group (Table 2, Fig. 1).

**Table 2:** Brain regions with significantly different global centralities in the D-AD group compared with the nD-AD and NC groups as identified via ANOVA analysis

	D-AD	nD-AD	NC	*p*
Global Efficiency	0.12 ± 0.01	0.12 ± 0.01	0.16 ± 0.02	< 0.001
Local Efficiency	0.17 ± 0.02	0.17 ± 0.02	0.24 ± 0.04	< 0.001

*D-AD*, AD with depression; *nD-AD,* non-depressed AD patients; *MNI*, Montreal Neurological Institute.

### Local properties

**Fig. 2:**
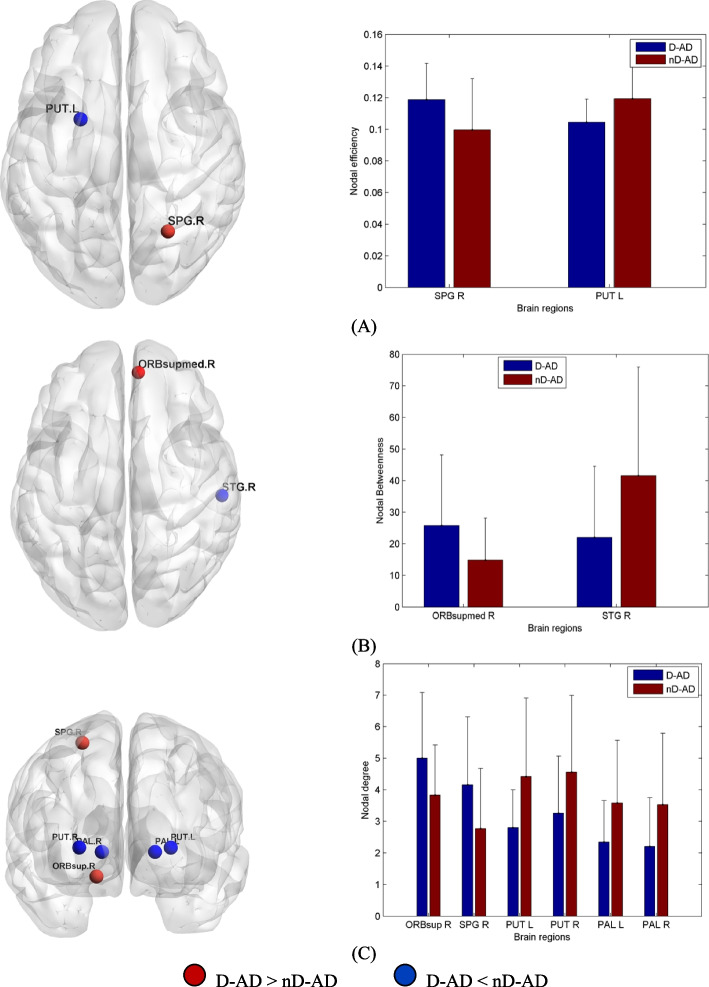
Brain areas showing significant group differences in nodal centrality. Red/blue represent higher/lower values of regional nodal centrality in D-AD patients for nodal efficiency (A), nodal betweenness (B), and nodal degree (C). In comparison with nD-AD patients, D-AD patients showed decreased nodal centrality in the pallidum, putamen, and right superior temporal gyrus. They also showed increased nodal centrality in the right superior parietal gyrus, right superior frontal gyrus, the medial orbital part of the right superior central gyrus, and the orbital part of the right superior frontal gyrus. PCG.R, right posterior cingulate gyrus; CAL.R, right calcarine gyrus; SPG.R, right superior parietal gyrus; STG.R, right superior temporal gyrus; ORBsupmed.R, medial orbital part of the right superior frontal gyrus; ORBsup.R, orbital part of the right superior frontal gyrus; PUT.L, left putamen; PUT.R, right putamen; nD-AD, non-depressed AD patients; D-AD, depressed AD patients

Compared with nD-AD patients, D-AD patients showed decreased nodal centrality in the pallidum, putamen, and right superior temporal gyrus. They also showed increased nodal centrality in the right superior parietal gyrus, right superior frontal gyrus, medial orbital part of the right superior frontal gyrus, and orbital part of the right superior frontal gyrus (Table 3, Fig. 2). We used the AAL template and REST (www.restfmri.net) to determine coordinates and size of the affected brain regions.

**Table 3:** Brain regions with significantly different nodal centrality in the D-AD group compared with the nD-AD group, identified via post hoc analysis

Brain regions	Voxels	MNI coordinates			*pcorr* value
		x	y	z	
Nodal Efficiency					
Right superior parietal gyrus	647	26	− 59	62	0.0245
Left putamen	306	−23	4	2	0.0223
Nodal Betweenness					
Right superior frontal gyrus/medial orbital	256	8	51	−7	0.0389
Right superior temporal gyrus	963	58	−21	7	0.0272
Nodal Degree					
Right superior frontal gyrus/orbital part	311	18	48	−14	0.0368
Right superior parietal gyrus	647	26	−59	62	0.0241
Left putamen	306	−23	4	2	0.0066
Right putamen	322	28	5	2	0.0484
Left pallidum	81	−18	0	0	0.0166
Right pallidum	76	21	0	0	0.0258

*D-AD*, AD with depression; *AD,* non-depressed AD patients; *MNI*, Montreal Neurological Institute. *Pcorr*, Bonferroni correction.

**Fig. 3:**
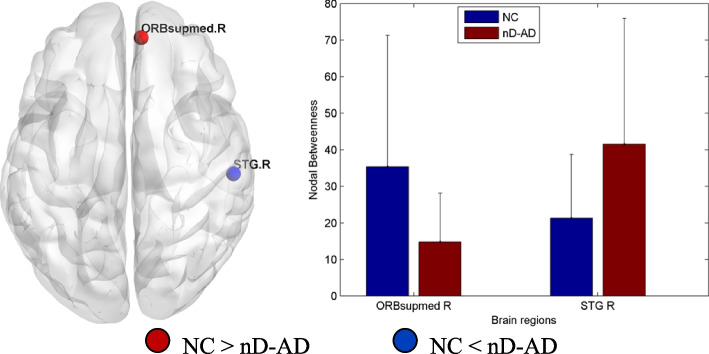
Brain areas showing significant group differences in nodal betweenness between nD-AD and NC groups. Blue/red represent higher/lower values of regional nodal betweenness in nD-AD patients. In comparison with nD-AD patients, NC showed decreased nodal betweenness in the right superior temporal gyrus and increased nodal betweenness in the medial orbital region of the right superior frontal gyrus. STG.R, right superior temporal gyrus; ORBsupmed.R, medial orbital part of the right superior frontal gyrus; nD-AD, non-depressed AD patients; NC, normal controls

Compared with nD-AD patients, NC showed decreased nodal betweenness in the right superior temporal gyrus and increased nodal betweenness in the medial orbital region of the right superior frontal gyrus (Table 4, Fig. 3).

**Table 4:** Brain regions with significantly different nodal centrality in the nD-AD group compared with the NC group, as identified via post hoc analysis

Brain regions	Voxels	MNI coordinates			*pcorr* value
		x	y	z	
Nodal Betweenness					
Right superior frontal gyrus/medial orbital	256	8	51	−7	0.0389
Right superior temporal gyrus	963	58	−21	7	0.0234

*nD-AD*, non-depressed AD patients; *NC*, normal controls; *MNI*, Montreal Neurological Institute. *Pcorr*, Bonferroni correction.

### Relationship between network parameters and clinical variables

We did not find any significant correlation between network parameters and clinical variables for any of the three groups.

## Discussion

In the current study, we examined alterations in the topological organization of functional brain networks in D-AD patients, nD-AD patients, and NC. D-AD and nD-AD groups show significantly reduced global and local efficiency compared with the NC group, in line with previous studies [27–29]. Li et al. reported that the NC group differed from pathological groups (early/late mild cognitive impairment and AD) with respect to global network properties, including transitivity, efficiency, and small world attribute. Efficiency was similar across AD and mild cognitive impairment (MCI) groups, but it differed between these groups and the NC group [29]. These results contribute useful insight into the neuropathological mechanisms underlying alterations of brain networks in AD patients, such as reduced information transfer efficiency, and resistance to random or targeted attacks [30].

We found no difference in the small-world attribute between D-AD/nD-AD patients and NC. We characterized small-world metrics using the speed of information transmission in the brain network and the degree of overlap between network nodes. We found inconsistent results for these metrics in AD. A study using 21 normal controls and 18 AD patients reported a reduction in the speed of information transmission for the AD group, but the degree of overlap between network nodes was not significantly different [17]. Another study using wavelets as a connectivity measure reported a reduction in the degree of overlap between network nodes for left and right hippocampus of AD patients, but the speed of information transmission was not significantly different [18]. Compared with controls, depressed adolescents present lower speed of information transmission and higher global efficiency [31], but no significant difference in the degree of overlap and local efficiency [32]. This result may be attributed to the fact that differences in these attributes reached significant levels across the three groups.

Compared with NC, nD-AD patients showed increased nodal centrality in the right superior temporal gyrus and decreased nodal centrality in the medial orbital portion of the right superior frontal gyrus. Compared with nD-AD, D-AD patients showed decreased nodal centrality in the pallidum, putamen, and right superior temporal gyrus (STG), and increased nodal centrality in the right superior parietal gyrus (SPG), /medial orbital portion of the right superior frontal gyrus (ORBsupmed), and right superior frontal gyrus (SFG). Our results suggest that alterations in the properties of the hub nodes may be characteristic network features of AD patients with depression. Pallidum and putamen are important structures of the basal ganglia [33]. Dysfunction of the basal ganglia is associated with inability to regulate reward behavior in depression and may be a potent precursor of MDD [34, 35]. Melancholic depressed participants present a smaller left putamen than non-melancholic depressed participants, and anhedonia has been associated with a smaller putamen [36]. Moreover, the severity of anhedonia has been associated with the FC of ventral caudate, cuneus, and middle temporal gyrus (MTG), suggesting the involvement of ventral caudate-cortical connectivity in anhedonia of MDD [29]. The reduced activity of caudate nucleus and STG also play a role in the neural mechanisms underlying decision-making in MDD [37]. Atrophy was evident in the ventral striatum, orbitofrontal cortex, and medial temporal lobe structures of MCI-AD and AD patients. Furthermore, the degree of atrophy in mesocorticolimbic regions positively correlated with the severity of depression, anxiety, and apathy in these patients [38]. Our results show decreased nodal centrality in pallidum, putamen, and right STG of D-AD. On the basis of these findings, we speculate that alterations in the connectivity of basal ganglia and temporal lobe may be related to depression in AD.

We also showed increased nodal centrality in the right SPG, left IFG, right SFG, right PCG, and right calcarine gyrus of D-AD patients. These brain regions are closely related to the parietal-limbic networks, frontoparietal network (FPN), and default-mode network (DMN), which play an important role in emotional regulation and cognitive appraisal. The parietal lobe and cingulate gyrus are involved in attentional, motivational, and emotional processes [39]. Therefore, abnormal connectivity may result in attentional bias and emotional restriction of MDD. Activity in the left anterior hippocampus/amygdala, subgenual cingulate, and medial prefrontal cortex decreased after psychotherapy in MDD patients, with associated improvement of depressive symptoms [40]. The global connectivity of FPN is also related to depressive symptoms [41]. Poor efficiency of FPN and DMN is associated not only with abnormal cognitive and executive functions, but also with characteristic depressive symptoms [42]. Emotion regulation, including expressive suppression and cognitive reappraisal, was closely related to the efficiency of FPN and DMN [43]. Furthermore, altered connectivity within these two networks may lead to emotional restriction caused by perception bias to negative emotions [44].

Our study presents some limitations. First, we found no correlation between network properties and clinical variables. This may be due to small sample sizes and inadequate clinical scales. Secondly, this study is a cross-sectional study, longitudinal was needed to observe changes in the topological properties of the AD network. Thirdly, diffusion tensor imaging has been used to study the structural network topological properties of AD. In the future, these properties should be studied using multimodal fMRI data [45–47]. Finally, we were not in a position to collect amyloid, Tau, or APOE information for this study. The absence of these indicators may affect the accuracy of patient enrolment.

## Conclusions

Our current study used a graph theory approach and rsfMRI to examine the topological organization of functional brain networks in D-AD and nD-AD patients. D-AD and nD-AD patients showed significantly lower global and local efficiency than NC. Some local brain regions were profoundly affected by D-AD. In particular, brain regions showing decreased node centrality were mostly located within the basal ganglia, while brain regions showing increased node centrality were mostly within the FPN. These findings may provide further insight into the neuropathophysiology underlying depression in AD.

## Supplementary information

**Additional file 1.**

## Data Availability

The datasets used and/or analyzed during the current study are available from the corresponding author on reasonable request.

## Abbreviations

AD: Alzheimer’s disease
D-AD: AD with depression
nD-AD: non-depressed AD patients
MMSE: Mini-Mental State Examination
D-NPI: depression domain on Neuropsychiatric Inventory
HAMD: Hamilton Depression Rating Scale
CDR: Clinical Dementia Rating
rsfMRI: resting-state functional MR imaging
