# Supplementary material for: Disrupted topological organization of functional brain networks in Alzheimer’s disease patients with depressive symptoms
Source: BMC Psychiatry. 2022 Dec 20;22:810. doi: 10.1186/s12888-022-04450-9 (PMC9764564; doi:10.1186/s12888-022-04450-9)
Supplement: Supplementary file 1 — Additional file 1. [file 12888_2022_4450_MOESM1_ESM.docx]

**Voxel-based morphometry analysis**

After correcting for image intensity nonuniformity, we used the most recent version of SPM8 (Wellcome Department of Imaging Neuroscience, London, UK) to divide MRI images into gray matter, white matter, and CSF. DARTEL (Wellcome Department of Imaging Neuroscience) was used to spatially normalize these segmented gray matter images in the standardized anatomic space to the customized template. We smoothed the images with an 8-mm FWHM Gaussian kernel and modulated them with Jacobean determinants derived from DARTEL's spatial normalization in order to preserve gray matter volume within each voxel. We first compared the grey matter density images of the three groups using ANOVA. We then used the difference brain areas obtained from ANOVA as a mask for post hoc analysis between the two groups using the two sample ttest. Multiple comparisons were corrected using FDR correction with a cluster p value < 0.05 and a voxel p < 0.005.

**Voxel-based morphometry results**

The ANOVA analysis demonstrated significant different gray matter density in a wide range of temporal lobe brain regions and hippocampus (Figure 1; Table 1). Compared with nD-AD, D-AD showed significant decline of gray matter density in the parahippocampal gyrus. Compared with NC, D-AD showed significant decline of gray matter density in the inferior temporal gyrus, left middle temporal gyrus, left superior temporal gyrus and right hippocampus; nD-AD showed significant decline of gray matter volume in the right inferior temporal gyrus and right hippocampus (Figure 2; Table 2).

**
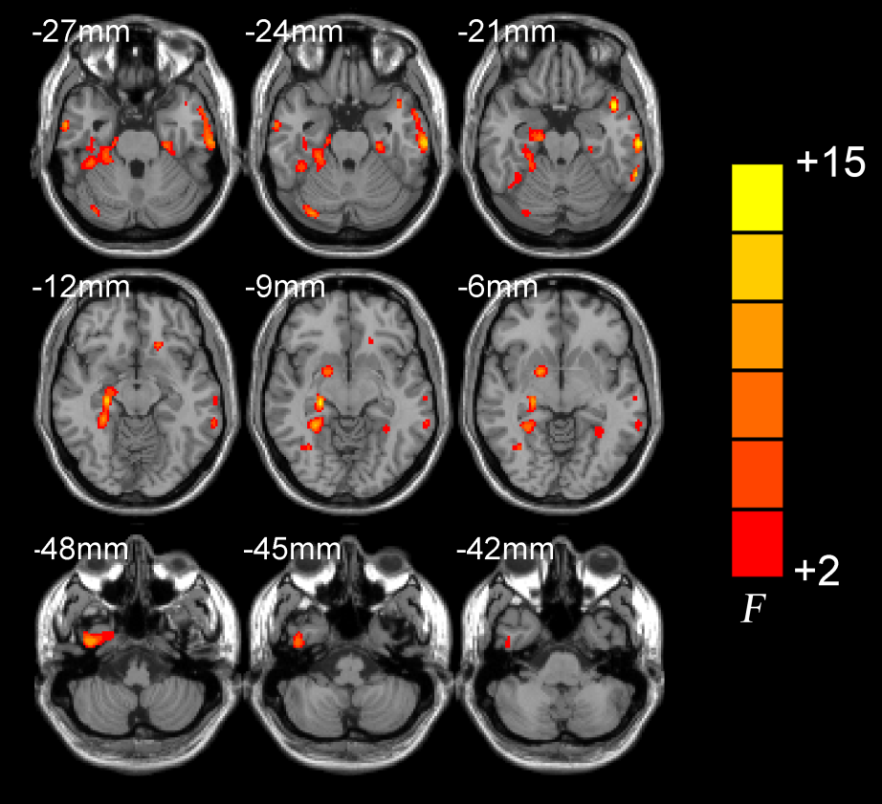
**

**Figure 1.** Brain regions with significantly different gray matter density in the D-AD group compared with the nD-AD and NC groups.

**Table 1.** Brain regions with significantly different gray matter density in the D-AD group compared with the nD-AD and NC groups

|  | **Brain regions** | **Voxels** | **MNI coordinates** | | | ***F*** |
| --- | --- | --- | --- | --- | --- | --- |
|  |  |  | **x** | **y** | **z** |  |
| Right inferior temporal gyrus | 55 | 40 | -2 | -48 | 7.4617 |  |
| Left middle temporal gyrus | 154 | -66 | -20 | -20 | 8.9925 |  |
| Left superior temporal gyrus | 43 | -44 | 14 | -20 | 9.0439 |  |
| Left inferior temporal gyrus | 25 | -64 | -48 | -20 | 8.1648 |  |
| Right hippocanpus | 113 | 26 | -24 | -10 | 8.7962 |  |
| Parahippocampa gyrus | 50 | 30 | -44 | -8 | 7.8076 |  |

*D-AD*, AD with depression; *nD-AD,* non-depressed AD patients; *NC*, normal controls; *MNI*, Montreal Neurological Institute.


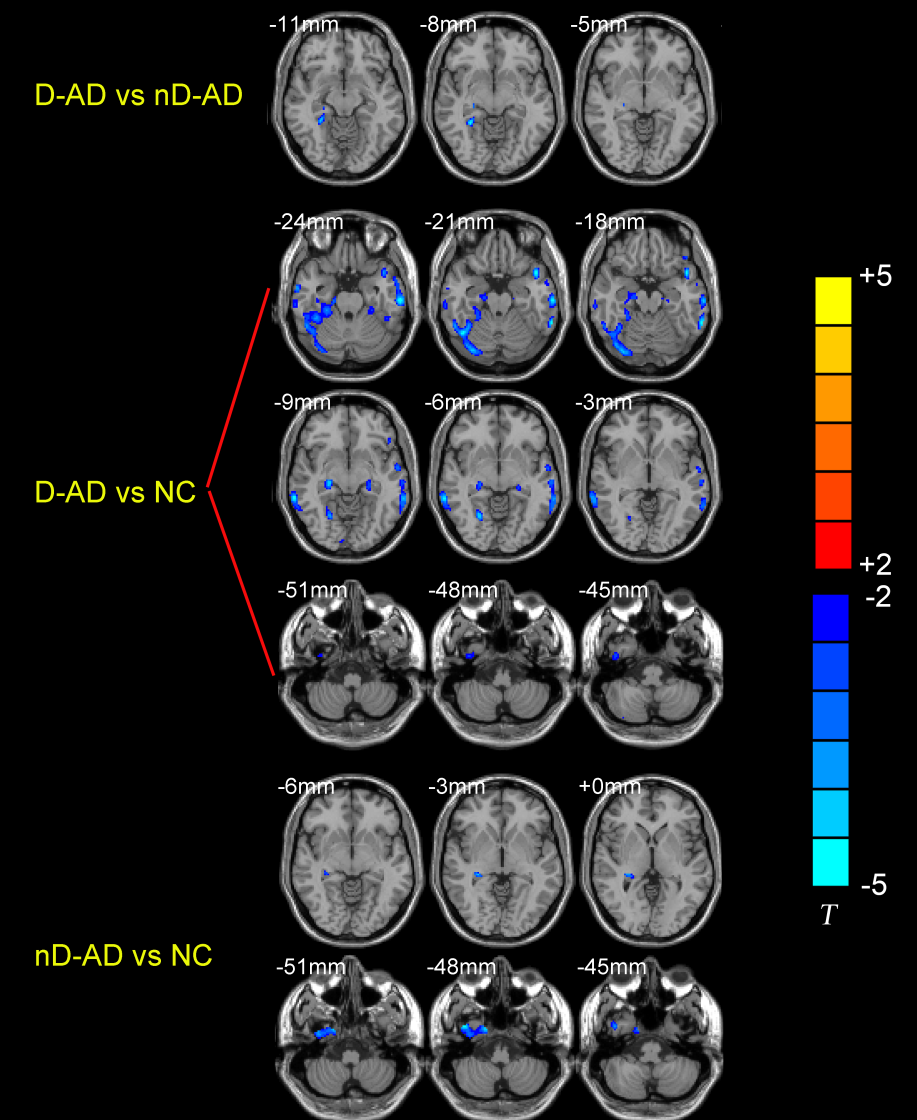


**Figure 2.** Brain regions with significantly different gray matter density in the D-AD group compared with the nD-AD and NC groups after post hoc analysis

**Table 2.** Brain regions with significantly different gray matter density in the D-AD group compared with the nD-AD and NC groups after post hoc analysis

|  | **Brain regions** | **Voxels** | **MNI coordinates** | | | ***T*** |
| --- | --- | --- | --- | --- | --- | --- |
|  |  |  | **x** | **y** | **z** |  |
| **D-AD vs nD-AD** |  |  |  |  |  |  |
| Parahippocampa gyrus | 87 | 30 | -44 | -8 | -3.5194 |  |
| **D-AD vs NC** |  |  |  |  |  |  |
| Left inferior temporal gyrus | 41 | -58 | -2 | -32 | -3.9818 |  |
| Right inferior temporal gyrus | 36 | 64 | -4 | -26 | -4.1392 |  |
| Left middle temporal gyrus | 136 | -64 | -20 | 22 | -4.6961 |  |
| Left superior temporal gyrus | 61 | -44 | 14 | -20 | -4.7195 |  |
| Right hippocampus | 57 | 26 | -24 | -10 | -4.4972 |  |
| **nD-AD vs NC** |  |  |  |  |  |  |
| Right inferior temporal gyrus | 244 | 44 | 6 | -48 | -4.4002 |  |
| Right hippocampus | 47 | 30 | -32 | -2 | -3.9303 |  |

*D-AD*, AD with depression; *nD-AD,* non-depressed AD patients; *NC*, normal controls; *MNI*, Montreal Neurological Institute.
